# Equity and spatial effects of hospital accessibility in Sichuan Province: evidence from GA2SFCA-based data

**DOI:** 10.3389/fpubh.2026.1950200

**Published:** 2026-10-12

**Authors:** Qianwen Song, Chengzhi Ge, Jing Deng

**Affiliations:** 1 Procurement Section of Shuangliu Maternal and Child Health Hospital, Chengdu, China; 2 Hefei Orthopaedics Hospital, Hefei, China; 3 Medical Equipment Department, West China Airport Hospital, Sichuan University (The First Peoples Hospital of Shuangliu District Chengdu), Chengdu, China

**Keywords:** hospital accessibility, Palma ratio, Spatial Durbin Model, spatial effects, spatial equity

## Abstract

**Introduction:**

Healthcare accessibility lies at the core of health equity; however, provincial-scale evidence on its spatial inequality and cross-county interactions remains limited, particularly in western China.

**Methods:**

We used a 2020 national 1 km grid accessibility dataset and aggregated scores to 183 county-level units. Equity was assessed using the coefficient of variation and Palma ratio across prefecture, county, and grid scales. Subsequently, we analyzed the spatial effects of accessibility using Moran’s index and a Spatial Durbin Model (SDM).

**Results:**

Accessibility exhibited a significant east-high, west-low gradient distribution. Inequality intensified with finer spatial units: the coefficient of variation increased from 42.16% at the prefecture level to 77.23% at the county level, and reached 148.28% at the grid level. Significant positive spatial autocorrelation confirmed clustering patterns, and the SDM spatial lag parameter was significantly positive. Direct effects showed that GDP and road density enhanced county-level accessibility, while fiscal expenditure and altitude exerted negative effects; population size had no significant direct effect. Indirect effects presented a different picture: population size had a negative spillover on neighboring areas, whereas fiscal expenditure generated positive cross-county spillovers.

**Conclusion:**

These findings indicate that healthcare planning in western China cannot rely solely on administrative boundaries. To reduce accessibility disparities, policies should prioritize differentiated resource allocation, coordinate transport and medical infrastructure planning, and refine fiscal transfer mechanisms to leverage positive spatial spillovers.

## 1 Introduction

Access to healthcare services is a core dimension of health equity, reflecting residents’ ability to obtain necessary diagnostic and treatment services within a reasonable time and at a reasonable travel cost. This measure accurately reflects the alignment between residents’ healthcare needs and the spatial supply of services (1), and serves as a key indicator for assessing the rationality of regional public health resource allocation and the equity of basic healthcare services (2). Therefore, examining the spatial equity of healthcare and public health services from an accessibility perspective is crucial for advancing the equalization of public health services and narrowing regional health resource disparities. The ‘Healthy China 2030’ Outline (3) explicitly identifies safeguarding health equity and narrowing regional and urban–rural disparities in healthcare services as key development principles. It proposes scientifically allocating regional healthcare resources according to the permanent resident population and service radii, as well as integrating and sharing healthcare resources through provincial-level coordination. These measures establish the overarching direction for spatially balanced resource allocation at the highest institutional level. Since 2021, primary healthcare institutions have consistently accounted for over 50% of total consultations in China, and more than 90% of residents can access healthcare services within a 15-min travel time. However, China continues to face a long-term concentration of healthcare resources in the more developed eastern provinces, while allocation in the western regions remains relatively scarce, resulting in substantial regional disparities. In the vast and sparsely populated western regions, primary healthcare institutions in plateau, mountainous, and ethnic minority areas confront practical challenges such as overly large service radii, severe transport barriers, and brain drain. Consequently, optimizing healthcare accessibility has become a key research priority.

Equity in healthcare access is widely regarded as the most important measure of opportunity equity. Accessibility can be understood as the ease with which people can reach desired locations and opportunities from a given point (4); it results from the interaction among residents, transport systems, and healthcare resources. Historically, early research on healthcare accessibility predominantly employed the doctor-to-patient ratio (5) or the shortest-distance method (6). The former overlooks spatial heterogeneity, while the latter disregards competition among facilities; neither accurately reflects real-world healthcare-seeking behavior. To address these shortcomings, Luo and Wang (1), building on the work of Radke et al. (7), formally proposed the two-step floating catchment area method. By incorporating the supply capacity of healthcare facilities, the distribution of residents’ demand, and spatial distance decay, this method overcomes the limitations of traditional simple ratio and shortest-distance approaches that ignore the spatial interaction between supply and demand. It has become the mainstream tool for spatial evaluation of healthcare services in health geography. In the early 21st century, the method was introduced into healthcare research in China and has yielded a wealth of findings. For example, Han et al. (8) found that accessibility to primary healthcare services in Tianjin declines from the city center to the periphery, with a significant shortage in the outer suburbs. Tao et al. (9) found that individuals reliant on public transport are at a distinct disadvantage when accessing healthcare. Xu et al. (10) demonstrated that, although overall healthcare accessibility coverage was high, marked disparities existed in resource distribution across prefecture-level cities. Yu et al. (11) found that the spatial distribution of general hospitals in Wuhan’s central urban area did not adequately meet the needs of different age groups. Gao et al. (12) identified hospital density, altitude, and the road network as the primary factors influencing healthcare access on the Qinghai-Tibet Plateau.

Although a wealth of research on healthcare access exists, regional research gaps persist. First, existing studies are geographically concentrated in the eastern and central regions, particularly the Yangtze River Delta, the Pearl River Delta, and Wuhan. While some research addresses the western regions, it largely focuses on Tibet, leaving Sichuan Province relatively underexplored. Second, the literature is predominantly case-based and focuses on municipal, county, or community levels; findings therefore mainly describe accessibility differentiation within small areas, while large-scale provincial-level studies remain scarce. Third, most studies consider only the direct local associations of various factors, overlooking spatial dependence and cross-regional interaction that may arise from resource flows and shared service catchments. Against this backdrop, this study addresses national strategic needs by systematically characterizing the spatial features of hospital accessibility in Sichuan. We use the coefficient of variation and the Palma ratio to examine spatial equity at multiple scales, prefecture, county, and 1 km grid. Spatial autocorrelation analysis is then used to identify clustering and the distribution of hotspots and coldspots. Finally, an SDM is used to characterize associations among natural, economic, demographic, and policy factors and hospital accessibility, including cross-county spatial dependence. Because the analysis is cross-sectional and the outcome is an externally derived accessibility measure, these results are treated as spatial associations rather than independently identified causal mechanisms. This study aims to deepen understanding of the spatial pattern of hospital accessibility in Sichuan and to provide a scientific basis for precise and equitable healthcare resource allocation in the province and the broader western region of China.

The contributions of this study are twofold. First, it provides empirical evidence on multi-scale spatial equity in hospital accessibility in Sichuan, a populous and highly heterogeneous western province that remains under-studied relative to eastern and central China. Second, it applies an SDM to county-level accessibility and provides provincial evidence on spatial dependence and cross-county associations, extending descriptive spatial mapping toward covariate-based analysis. These contributions are empirical and regional in nature rather than methodological innovations.

## 2 Data and methods

### 2.1 Study area

As shown in Figure 1, Sichuan Province is located in the heart of southwestern China, between 97°21′ and 108°33′ east longitude and 26°03′ and 34°19′ north latitude. It covers a total administrative area of 486,100 km^2^ and comprises 21 prefecture-level divisions (including cities and autonomous prefectures) and 183 county-level divisions (counties, county-level cities, and districts). The province spans five major topographical units: the Qinghai-Tibet Plateau, the Hengduan Mountains, the Yunnan-Guizhou Plateau, the Qinba Mountains, and the Sichuan Basin. The terrain generally slopes from west to east, with significant variations in elevation; the average altitude across the province is 2,598 meters. Specifically, the eastern basin region has an average altitude of less than 800 m, with gentle terrain and a high population concentration, while the western Sichuan Plateau region has an average altitude exceeding 3,200 m and is characterized by widespread high mountains and gorges. Mountainous areas account for 79.52 per cent of the total area, with a sparse road network and low population density. As shown in Figure 1, based on economic geography, the province can be divided into five major economic regions: the Chengdu Plain Economic Zone, the Southern Sichuan Economic Zone, the Northeastern Sichuan Economic Zone, the Panxi Economic Zone, and the Northwestern Sichuan Ecological Demonstration Zone. These regions differ significantly in economic development, transport infrastructure, fiscal capacity, and population density. Furthermore, Sichuan Province contains three ethnic minority autonomous prefectures—Aba, Ganzi, and Liangshan—with a large ethnic minority population, making it a key area of ethnic minority settlement in southwestern China.

**Figure 1:**
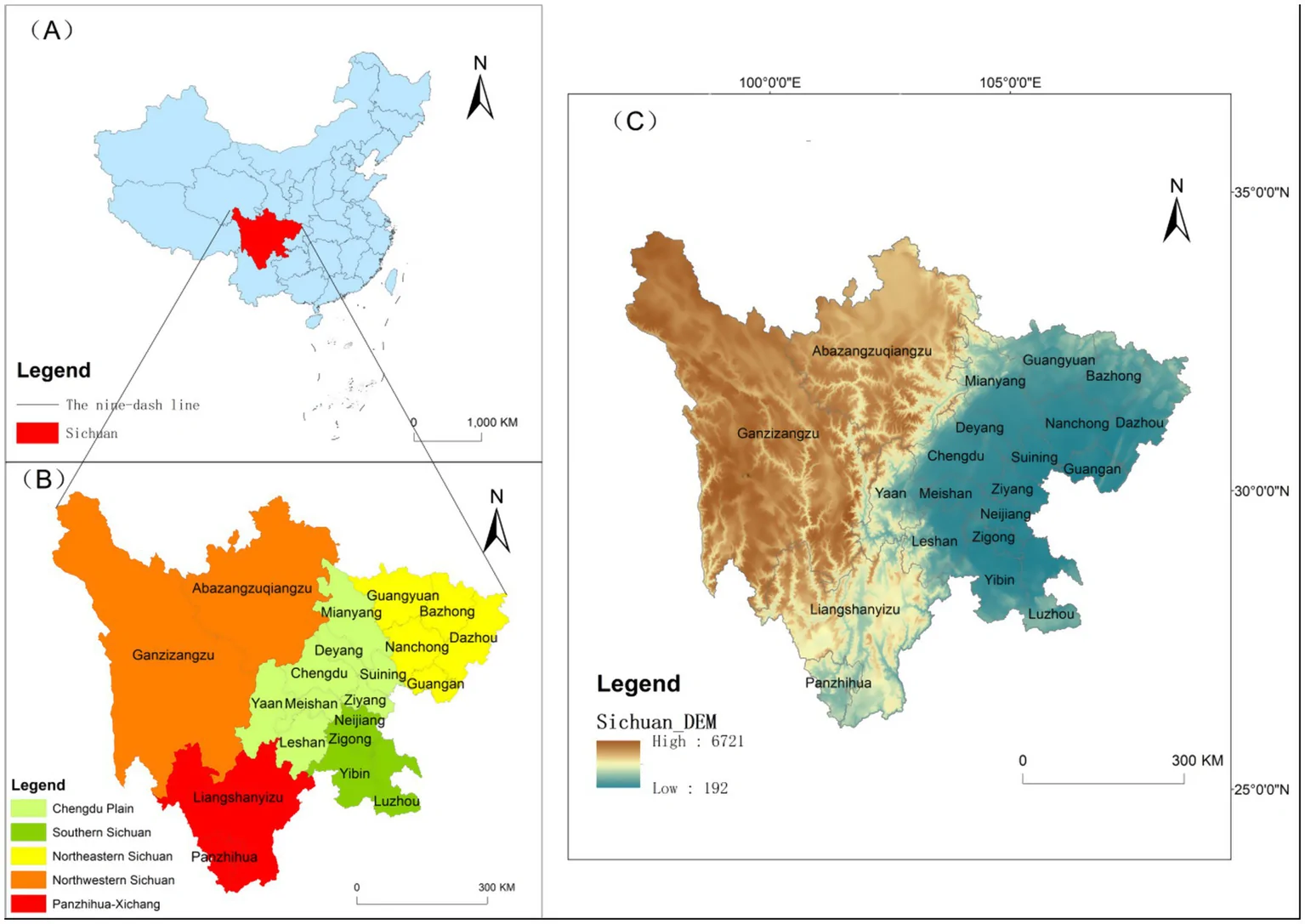
Map showing the location of Sichuan Province. **(A)** Location map of Sichuan Province; **(B)** map of the five major economic regions in Sichuan Province; **(C)** Sichuan Province topographic map. Reproduced or adapted from Ye et al. (13). License: CC BY 4.0 (http://creativecommons.org/licenses/by/4.0/). Source: Figshare, doi: 10.6084/m9.figshare.c.7318976.v4.

### 2.2 Data sources

This study uses an externally derived measure of hospital accessibility rather than calculating the measure anew. The source indicator is the nationwide 1 km x 1 km grid-based hospital accessibility dataset for China reported by Ye et al. (13), with 2020 as the base year. To avoid ambiguity, we distinguish the construction of that source dataset from the analyses performed in the present study. In the source dataset, road-network travel time was estimated from settlements, represented by grid cells, to hospitals using OpenStreetMap road data and a Contraction Hierarchies pathfinding algorithm; hospital locations and bed capacity were compiled from the YAOZH medical data platform; and population demand was drawn from WorldPop at approximately 1 km resolution. Hospital accessibility was then computed with the Gaussian two-step floating catchment area (GA2SFCA) method. In the first step, each hospital’s bed supply is divided by the population within a tier-specific catchment threshold, set at 30, 45, and 60 min for primary, secondary, and tertiary hospitals, respectively. In the second step, for each grid cell, the supply-to-demand ratios of hospitals within these thresholds are summed after applying a Gaussian distance-decay weight. The resulting grid value is therefore a relative measure of hospital bed capacity available to the local population after distance decay; higher scores indicate greater hospital supply relative to population within reachable catchments. It is not a travel-time indicator itself. We used these accessibility scores directly as the outcome in the equity and spatial-econometric analyses and did not recalibrate any GA2SFCA parameter. The digital elevation model (DEM) data were obtained from the Geospatial Data Cloud.^1^ County-level socioeconomic variables were derived from the 2021 China County Statistical Yearbook, whose statistics refer to the 2020 calendar year and are therefore temporally consistent with the accessibility data. This score is designed to capture effective healthcare accessibility, which arises from the interaction of service supply, population demand, and distance decay, rather than geographical proximity alone. Two locations with similar travel times can thus differ considerably in effective accessibility when supply or demand is unevenly distributed. Accordingly, we do not treat the indicator as a pure proximity or travel-time measure.

At the grid scale, the 1 km accessibility scores were used directly. For county- and prefecture-level summaries and regressions, grid values were aggregated to each administrative unit using unweighted zonal arithmetic means of grid values within the unit. Population weights were not applied at the aggregation stage or in the equity indicators. The equity measures therefore compare spatial units with equal statistical weight and describe territorial inequality across places rather than inequality experienced by resident populations. The SDM and all spatial analyses were estimated on the 183 county-level units.

### 2.3 Research methods

#### 2.3.1 Coefficient of variation

This study employs the coefficient of variation to measure spatial imbalance in hospital accessibility across 1 km × 1 km grids, counties, and cities; a higher value indicates greater relative disparity. The formula is as follows:

$$\mathrm{CV}=\frac{\sigma }{\bar{x}}$$ (1)

In Equation 1, CV denotes the coefficient of variation; sigma denotes the standard deviation of hospital accessibility; and x-bar denotes the arithmetic mean of hospital accessibility, each calculated for the respective spatial scale (1 km x 1 km grid, county, or prefecture-level unit).

#### 2.3.2 Palma ratio

Tao et al. (14) argued that the coefficient of variation and the Palma ratio are effective measures of spatial equity in healthcare facilities. To address limitations of the Gini coefficient and the Theil index, which are overly sensitive to changes in the middle of the distribution, this study uses the Palma ratio as a primary indicator of inequality in county-level hospital accessibility and computes the same ratio at prefecture and 1 km grid levels. Originally proposed by Chilean economist José Gabriel Palma (15) to analyze income inequality, the Palma ratio emphasizes the contrast between the top and bottom of the distribution rather than its middle. This conceptual focus is equally pertinent to healthcare accessibility, where policy concern often centers on the polarization between well-served and poorly served places. Accordingly, the ratio is adapted here in a descriptive manner: at each spatial scale, units are ranked by accessibility; the numerator is the cumulative accessibility of the top 10% of units and the denominator is the cumulative accessibility of the bottom 40%. Because each spatial unit receives equal statistical weight, the measure explicitly describes polarization across places rather than across populations. This unweighted choice is intentional: our interest lies in territorial equity across administrative units and grid cells—the level at which resource allocation decisions are typically made. A population-weighted variant would instead represent inequality as experienced by residents, and could be considered in future analyses. As shown in Equation 2:

$$\text{Palma Ratio}=\frac{\mathrm{Sum}\;\text{of accessibility of the}\;\mathrm{top}\;10\%\text{units}}{\text{Total accessibility of the bottom}\;40\%\text{units}}$$ (2)

#### 2.3.3 Methods for analyzing spatial distribution

Spatial autocorrelation analysis is commonly used to examine the interdependence and spatial clustering of variables distributed across the same area (16). This study employs the Global Moran’s I and the Local Indicators of Spatial Association (LISA) index to assess the spatial correlation and clustering of hospital accessibility in Sichuan Province.

#### 2.3.4 Global Moran’s I

Global Moran’s I measures the spatial autocorrelation of a variable. A statistically significant positive value indicates a clustered distribution, while a significant negative value indicates a dispersed distribution. The magnitude of the index reflects the strength of spatial association: a value close to 1 indicates strong clustering, while a value close to −1 indicates strong dispersion (17). The formula is as follows:

$$\text{Mora}n^{’}s\;\;I=\frac{\sum_{i=1}^{n}\sum_{j=1}^{n}(X_{i}-\bar{X})(X_{j}-\bar{X})}{S^{2}\sum_{i=1}^{n}\sum_{j=1}^{n}W_{\mathrm{ij}}}$$ (3)

In Equation 3, $X_{i}$ and $X_{j}$ denote the observed attribute values for regions i and j, respectively, while $W_{\mathrm{ij}}$ represents the corresponding element of the spatial weight matrix. The Moran’s I statistic ranges from −1 to 1. A value of I > 0 signifies positive spatial autocorrelation, indicating significant clustering in hospital accessibility; proximity to 1 implies stronger positive autocorrelation. Conversely, I < 0 denotes negative spatial autocorrelation, reflecting spatial dispersion in hospital accessibility, with values approaching −1 indicating stronger negative autocorrelation. Finally, I = 0 suggests the absence of significant spatial autocorrelation.

#### 2.3.5 LISA clustering

To identify local patterns of accessibility and their spatial agglomeration across Sichuan, this study implements Local Indicators of Spatial Association (LISA). In contrast to Global Moran’s I, which summarizes the overall degree of spatial dependency in the study region, LISA quantifies localized associations between individual county-level units and their neighbors and classifies units into High-High, Low-Low, High-Low, and Low-High categories (18).

$$I=\frac{\left(X_{i}-\bar{X}\right)}{S^{2}}\sum_{j}W_{\mathrm{ij}}(X_{j}-\bar{X})$$ (4)

In Equation 4, I denotes Moran’s index; $X_{i}$ and $X_{j}$ represent the hospital accessibility scores for counties i and j, respectively; $\bar{X}$ signifies the mean accessibility score across all counties in Sichuan Province; $W_{\mathrm{ij}}$ corresponds to the spatial weight matrix element, for which a binary contiguity matrix is adopted in this study; and S represents the sum of all elements within said matrix.

#### 2.3.6 Spatial Durbin Model

Hospital accessibility across county-level units in Sichuan is shaped not only by local characteristics such as medical service provision and socioeconomic conditions but may also be related to conditions in neighboring counties through capital and labor flows, environmental constraints, and transport infrastructure. This motivates explicit treatment of potential spatial dependence in accessibility. Accordingly, this study adopts a spatial econometric framework that accounts for spatial dependence and includes spatially lagged dependent and explanatory variables. Relative to conventional regression approaches, spatial econometric models formalize Tobler’s first law of geography, which states that nearer entities exhibit greater similarity, and thereby capture spatial autocorrelation in georeferenced data. By integrating spatial structure into the analysis, the framework mitigates estimation bias arising from omitted spatial effects (19). The specification is formalized as follows:

$$S_{i}=\alpha_{0}+\rho W_{n}S_{i}+\beta_{1}{Ζ}_{i}+\beta_{2}W_{n}{Ζ}_{i}+\varepsilon_{i}\;\;\;$$ (5)

In Equation 5, $S_{i}$ represents the hospital accessibility of county i, $W_{n}$ is the spatial weight matrix, and $W_{n}S_{i}$ is the spatial lag term of county-level hospital accessibility in neighboring counties; ${Ζ}_{i}$ is the control variable, $W_{n}{Ζ}_{i}$ is the spatial lag term of the influencing variable; $\varepsilon_{i}$ is the error term, and, is the spatial autocorrelation coefficient, is the influence coefficient of each control variable, and is the coefficient of the spatial lag term.

In this specification, i indexes county-level units in Sichuan Province, and the dependent variable is county-level hospital accessibility. The spatial weight matrix W is defined as a binary first-order queen contiguity matrix. This choice is motivated by three considerations. First, county boundaries in Sichuan largely coincide with the jurisdictional scope of health resource allocation, so cross-county patient flows and shared service catchments are most likely to occur between adjacent counties. Second, the queen criterion captures both shared edges and vertices, thus providing a more complete representation of potential spatial interactions than the rook criterion. Third, this specification is conventional in county-level spatial equity research, which facilitates comparability with previous studies (20). W is intentionally left unstandardized. As a result, the spatial lag terms are sums of neighboring values rather than averages over neighboring counties. This interpretation is consistent with our impact decomposition, where spatial lags are treated as the cumulative total effect of all adjacent counties.

#### 2.3.7 Explanatory variables for the Spatial Durbin Model

Drawing on the literature and recognizing the heterogeneous developmental stages of Sichuan’s county-level jurisdictions, we examine five county-level covariates. Economic development is proxied by gross domestic product (GDP) (20). Government intervention is operationalized as total local government fiscal expenditure (GM) (21). Demographic scale is measured by total permanent resident population (POP) (22). Transport infrastructure is quantified by road network density (Trf) (23). Topographic constraints are accounted for by mean county-level elevation (ALT) (24). All covariates correspond to the 2020 reference year of the accessibility data. For the spatial econometric analysis, the outcome and all five covariates were used in natural-logarithm form, so coefficients approximate elasticities. Before estimation, multicollinearity was checked with variance inflation factors (VIF) from the OLS specification: 7.22 for population, 3.66 for fiscal expenditure, 3.22 for altitude, 1.75 for road density, and 1.64 for GDP (mean VIF = 3.50); all values were below 10, indicating no severe multicollinearity.

## 3 Results

Hospital accessibility in Sichuan Province was classified into five categories using the natural breakpoint method: low, relatively low, moderate, relatively high, and high accessibility (see Figure 2). Overall, hospital accessibility exhibits a distinct spatial pattern of higher accessibility in the east and lower in the west, and higher in the plains and lower on the plateaus. With the Panzhihua–Ya’an–Guangyuan corridor serving as a dividing line, hospital accessibility in the eastern region is markedly superior to that in the western region. Specifically, the western region is characterized predominantly by low or relatively low accessibility, shown as extensive contiguous dark green areas. In contrast, the eastern region features a dense distribution of grids with high or relatively high accessibility, forming red and orange clusters centered on Chengdu. Distinct high-value zones are also evident in cities such as Zigong, Neijiang, and Yibin in southern Sichuan, as well as in areas bordering Chongqing and in Guang’an in southeastern Sichuan; county seats in the eastern region consistently serve as the centers of these high-accessibility zones.

**Figure 2:**
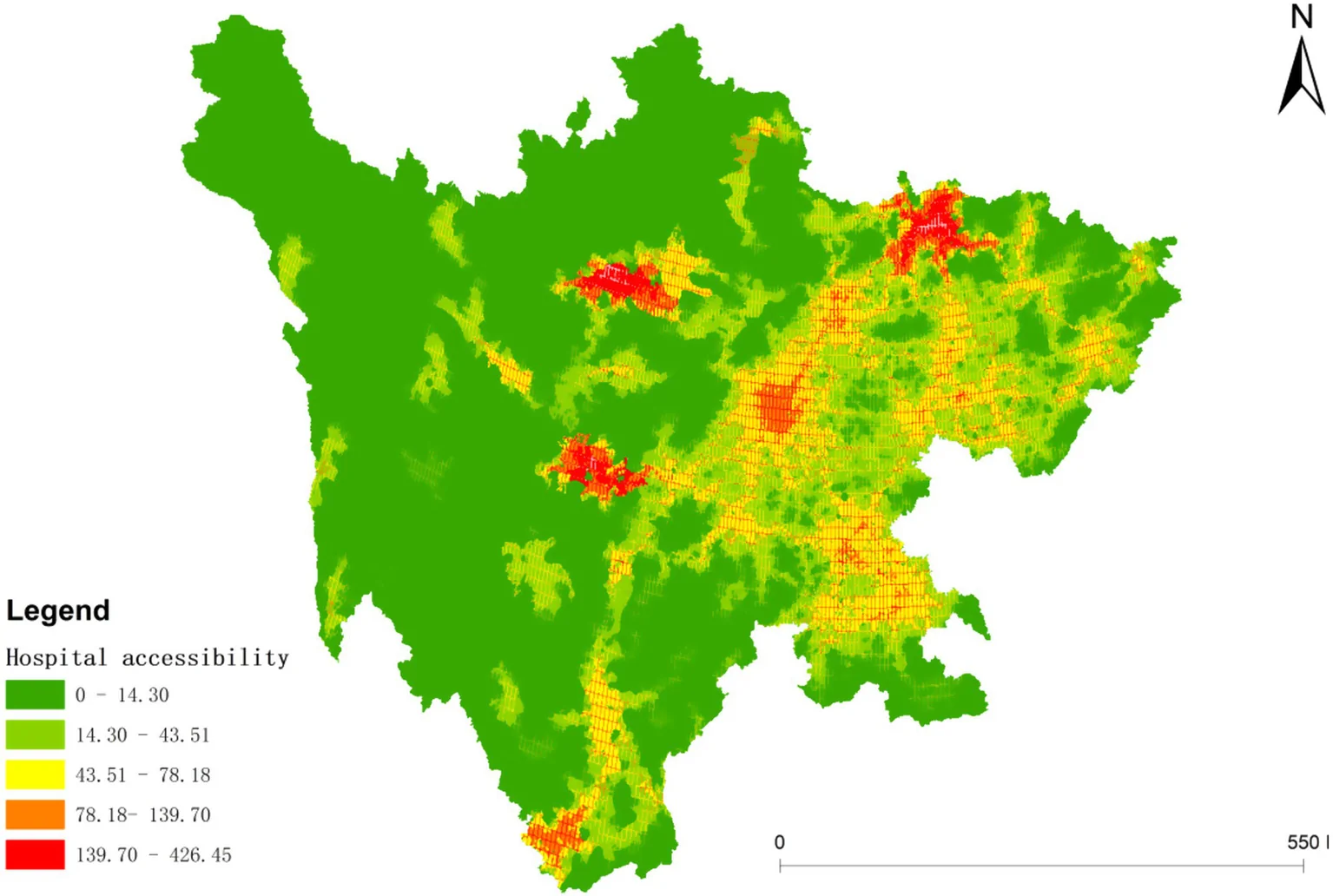
Spatial distribution of accessibility to hospitals in Sichuan.

Second, areas with low or moderate accessibility account for the vast majority of the province’s total area and are primarily distributed across the Western Sichuan Plateau and its surrounding regions. These areas face challenges including sparse population, complex topography, and poor transport infrastructure, which hinder effective healthcare coverage. Healthcare resources within these regions are concentrated in a few key towns such as Barkam and Kangding, leaving vast remote rural and pastoral areas with limited convenient access, further exacerbating the overall low accessibility. In contrast, grids with high and relatively high accessibility are highly concentrated in the core cities of the basin—including Chengdu, Deyang, Mianyang, Meishan, and Ziyang—and their surrounding plains, demonstrating a pronounced centralization effect. This distribution pattern is primarily driven by topography and economic development. The Chengdu Plain, with its flat terrain, dense road network, and concentration of high-quality healthcare resources, has consequently achieved high hospital accessibility.

From a regional perspective (Table 1), hospital accessibility across Sichuan’s five major economic zones reveals substantial spatial heterogeneity. Mean accessibility ranks in descending order as follows: the Chengdu Plain Economic Zone, the Southern Sichuan Economic Zone, the Panxi Economic Zone, the North-Eastern Sichuan Economic Zone, and the North-Western Sichuan Ecological Demonstration Zone. Although the Chengdu Plain Economic Zone records the highest mean accessibility, a distinct core-periphery structure prevails. This pattern is consistent with the concentration and radiation dynamics of the provincial capital, Chengdu, and significant intra-regional disparities persist even among counties near the provincial core. In contrast, the Southern Sichuan Economic Zone shows the most balanced development, with relatively uniform coverage of healthcare resources across major population centers. The Panxi Economic Zone occupies a middle rank and is internally divided: Panzhihua City has high accessibility attributable to its industrial base, whereas Liangshan Prefecture has more limited resources and lower accessibility, exacerbated by topography. Notably, the North-Eastern Sichuan Economic Zone registers the highest peak accessibility, but this anomaly likely stems from an upward bias of the GA2SFCA approach in the Guangyuan region, where concentrated supply meets sparse demand, rather than necessarily reflecting genuine service capacity. Delamater (25) cautions that 2SFCA-type accessibility measures can overestimate accessibility in such contexts, and analogous risks may occur in Ganzi and Aba. Finally, the North-Western Sichuan Ecological Demonstration Zone exhibits critically low accessibility, constrained by an inhospitable alpine environment, extreme climatic conditions, demographic sparsity, and underdevelopment.

**Table 1:** Statistical analysis of hospital accessibility across different regions of Sichuan Province.

Economic Zone	Mean	Min	Max	SD
Chengdu Plain Economic Zone	38.75	5.95	91.89	22.53
The Economic Zone of Northeastern Sichuan	35.37	8.05	149.22	26.05
Southern Sichuan Economic Zone	36.01	4.11	66.84	19.53
The Ecological Demonstration Area of Western Sichuan	12.88	0.00	72.13	15.91
Panxi Economic Zone	21.93	3.17	95.21	26.42

### 3.1 Analysis of accessibility equity at different spatial levels

The coefficient of variation (CV) quantifies the extent of dispersion in healthcare accessibility relative to the mean and is used to compare distributional equity across spatial scales (26). As shown in Table 2, the CV is 42.16% at the prefecture level, 77.23% at the county level, and 148.28% at the 1 km x 1 km grid level. These values show that measured inequality increases as the spatial unit becomes finer. Part of this pattern is expected from spatial aggregation and the modifiable areal unit problem (MAUP): averaging heterogeneous grid values within counties and prefectures smooths within-unit variation, so coarser scales necessarily display less measured inequality. The estimates should therefore not be read solely as evidence that smaller-scale variation is intrinsically more inequitable. No population weighting was applied, so these values describe inequality across spatial units rather than inequality experienced by residents. The pronounced east–west dichotomy remains a substantive contributor, while the agglomeration of human and financial capital in economically advantaged eastern areas reinforces the imbalance, but the aggregation mechanism contributes mechanically as well.

**Table 2:** Analysis of equity in access to hospitals in Sichuan Province.

Type	CV	Palma ratio
Prefecture-level	42.16%	1.05
County	77.23%	2.09
Grid	148.28%	3.97

The Palma ratio follows the same scale pattern, standing at 1.05 at the prefecture level, 2.09 at the county level, and 3.97 at the grid level. A value near 1 means that high- and low-accessibility units have broadly comparable cumulative scores, whereas a value of 3.97 means that the top 10% of grid cells possess nearly four times the cumulative accessibility of the bottom 40%. Because population weighting was not used, these ratios quantify polarization across places. The comparison is therefore meaningful for territorial allocation but does not directly measure population-experienced inequality.

In summary, inter-prefecture disparities in hospital accessibility across Sichuan are modest, county-level disparities are larger, and grid-level disparities are the most pronounced. The widening pattern is amplified by aggregation and MAUP, and at every scale the indicators describe inequality across spatial units rather than across residents. Finer scales remain valuable because they reveal service blind spots that coarse aggregates obscure.

### 3.2 Analysis of spatial characteristics

To further characterize the spatial configuration of hospital accessibility, we implemented global spatial autocorrelation analysis for the 183 county-level units in 2020. As Table 3 indicates, Moran’s I is statistically significant and positive, confirming that county-level accessibility is spatially clustered rather than randomly distributed.

**Table 3:** The Moran’s Index of hospital accessibility in Sichuan Province.

Year	Moran’s I	Z-value	*p*-value
2020	0.54	12.43	0.00

Global Moran’s I establishes the presence of county-level spatial clustering but does not reveal where clusters occur or how internally heterogeneous they are. We therefore applied LISA and mapped statistically significant High-High and Low-Low counties as hotspots and coldspots (Figure 3). Hotspots are predominantly located in the eastern sector, encompassing the Chengdu Plain and the Southern Sichuan Economic Zone, with additional nodes such as Guangyuan and Panzhihua. Coldspots form contiguous areas across the Western Sichuan Plateau and minority-concentrated regions including Liangshan. This east–west contrast is consistent with flatter terrain, denser road networks, higher economic development, and the agglomeration of major medical institutions in eastern Sichuan, and with weak economic foundations, sparse population, high altitude, and climatic constraints in the west.

**Figure 3:**
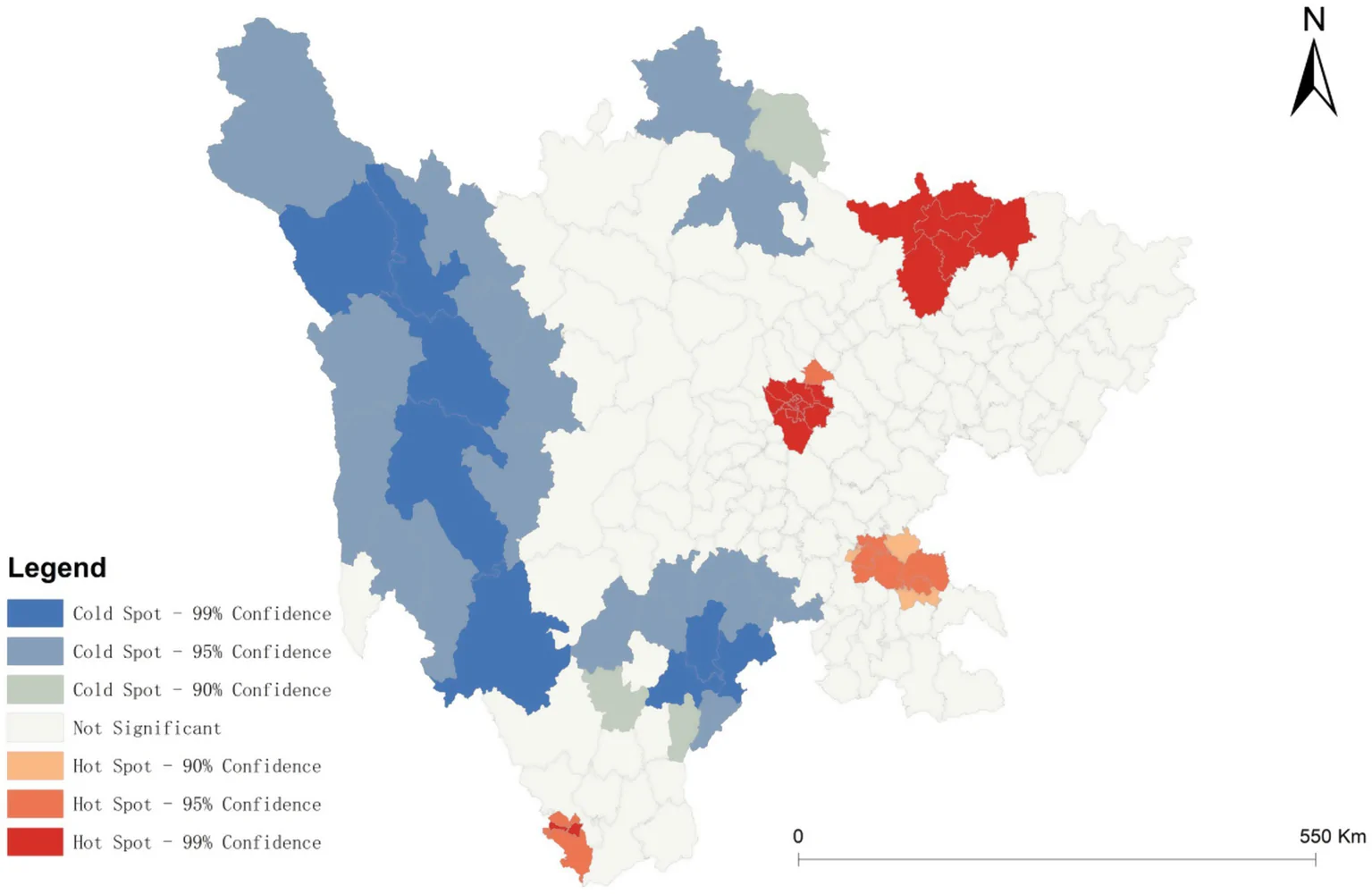
LISA-based hotspot and coldspot map of hospital accessibility in Sichuan (statistically significant county-level High-High/Low-Low clusters at 90, 95, and 99% confidence levels).

### 3.3 Spatial Durbin Model results

To identify the optimal spatial econometric specification, this study executed a sequential series of model selection diagnostics. Table 4 demonstrates that the global Moran’s I index is statistically significant and positive, validating the presence of positive spatial dependence in hospital accessibility. Results from both the Lagrange Multiplier Error and Lagrange Multiplier Lag tests yielded high significance levels. Consistent with the methodological prescriptions of Anselin and Florax et al. (27), the Spatial Durbin Model represents the most appropriate specification. Furthermore, the Likelihood Ratio test confirmed that the SDM significantly outperformed the ordinary least squares benchmark. Crucially, the joint significance test for the spatial lags of explanatory variables rejected the null hypothesis that these coefficients were jointly zero. This outcome effectively precludes the possibility of the SDM degenerating into a Spatial Autoregressive or Spatial Error model. Consequently, the Spatial Durbin Model was adopted as the primary analytical framework for this investigation.

**Table 4:** Results of the Spatial Durbin Model test.

TYPE	Value	*p*-value
LM-Lag test	14.52	0.00
Robust LM-Lag test	1867.69	0.00
LM-Error test	26.92	0.00
Robust LM-Error test	1880.09	0.00
LR-Lag test	44.23	0.00
LR Test (wX’s = 0)	34.25	0.00

Table 5 reports the estimated SDM coefficients. The estimated spatial autoregressive coefficient is positive and statistically significant with rho equal to 0.07 and z equal to 6.65, indicating spatial dependence or spatial feedback in county-level hospital accessibility under the specified model. GDP, road density, fiscal expenditure, and altitude each show statistically significant local coefficients, while the local coefficient of population is positive but only marginally significant. Among the spatial-lag coefficients, the lags of population, GDP, and fiscal expenditure are statistically significant, whereas the spatial lags of road density and altitude are not.

**Table 5:** Results of the Spatial Durbin Model.

Variable	Coeff	z-value	*p*-value
Trf	0.13	3.07	0.00
POP	0.21	1.94	0.05
GDP	0.70	6.35	0.00
GM	−0.42	−2.93	0.00
ALT	−0.40	−2.93	0.00
WxTrf	0.01	0.11	0.92
WxPOP	−0.11	−3.06	0.00
WxGDP	−0.07	−2.87	0.00
WxGM	0.14	3.19	0.00
WxALT	0.02	0.61	0.54
cons	1.33	0.68	0.50
ρ	0.07	6.65	0.00
R2	0.86		
*N*	183		

### 3.4 Spatial Durbin Model effect decomposition

To interpret the SDM estimates in terms of spatial associations, this study summarizes each explanatory variable through direct, indirect, and total effects. A direct effect captures the association between a variable and accessibility in the same county after the spatial feedback structure of the model is accounted for. An indirect effect captures the association transmitted to accessibility in neighboring counties through spatial dependence. The total effect is the sum of the direct and indirect components. Table 6 reports the average direct, indirect, and total effects together with delta-method z statistics.

**Table 6:** Direct, indirect, and total effects of explanatory variables on hospital accessibility.

Variable	Direct effect	Indirect effect	Total effect
Trf (road density)	0.137*** (3.34)	0.095 (1.12)	0.233*** (2.89)
POP (population)	0.164 (1.56)	−0.795*** (−2.60)	−0.630** (−1.98)
GDP	0.695*** (6.25)	−0.145 (−0.78)	0.550** (2.46)
GM (fiscal expenditure)	−0.369*** (−2.67)	0.939*** (2.63)	0.570 (1.61)
ALT (mean elevation)	−0.411*** (−3.09)	−0.116 (−0.68)	−0.528*** (−3.38)

Effects are computed from the SDM coefficients as average impacts of S_k(W) = (I-rho W)^-1(beta_k I + theta_k W), with delta-method z statistics in parentheses. ***p < 0.01, **p < 0.05.

From the perspective of economic development measured by GDP, GDP has the largest positive direct effect. Its direct effect equals 0.695 and passes the 1% significance test, indicating that a higher level of economic development in a county is associated with higher hospital accessibility in the same county. Higher economic development gives local governments stronger financial capacity to allocate medical facilities and equipment and gives residents greater ability to pay for care, both of which support accessibility (28). The indirect effect of GDP equals −0.145 and does not pass the significance test, so the decomposition provides no statistically significant evidence that neighboring economic development lowers local accessibility, although the spatial-lag coefficient of GDP is negative and significant in Table 5. However, economic advantages may still reduce the utilization of medical facilities in surrounding areas by attracting medical personnel and patients from those areas (29).

Population size has a positive direct effect of 0.164 that does not pass the significance test. In contrast, its indirect effect equals −0.795 and passes the 1% significance test, indicating that population concentration in one county is associated with lower accessibility in neighboring counties. This negative indirect association is consistent with China’s practice of allocating health facilities according to service population quotas. Counties with larger populations receive more health infrastructure and tend to attract additional investment beyond basic services, leaving fewer resources for surrounding counties and reducing their accessibility (30). Because the negative indirect component outweighs the positive direct component, the total effect equals −0.630 and passes the 5% significance test.

Road network density has a positive direct effect of 0.137 that passes the 1% significance test, indicating that a denser local road network is associated with higher local accessibility. A denser road network shortens the travel time from residential areas to medical institutions and thereby raises accessibility (31). The indirect effect equals 0.095 and is not statistically significant, while the total effect equals 0.233 and passes the 1% significance test. Road infrastructure therefore supports accessibility mainly through the direct component.

Fiscal expenditure has a direct effect equal to −0.369 that passes the 1% significance test. The negative sign does not imply that public spending is harmful to accessibility. Counties with high fiscal expenditure relative to GDP are often transfer-dependent underdeveloped counties whose expenditure structure is oriented away from health care and whose initial medical resources are limited (32). The indirect effect of fiscal expenditure equals 0.939 and passes the 1% significance test. Medical services financed in one county can spill over administrative borders, so residents of surrounding counties may benefit from those services and obtain higher accessibility (33). The positive indirect component offsets the negative direct component, leaving a positive total effect of 0.570 that does not reach statistical significance.

Altitude has a direct effect equal to −0.411 that passes the 1% significance test, indicating that higher mean elevation is associated with lower accessibility in the same county. This negative association is consistent with Liu et al. (34): high-altitude areas have complex terrain, constrained transport conditions, and dispersed populations, so medical facilities cover smaller service radii and residents face substantially higher travel costs than residents in lowland areas. The indirect effect equals −0.116 and is not statistically significant, while the total effect equals −0.528 and passes the 1% significance test.

Taken together, the decomposition results suggest that county-level hospital accessibility is shaped jointly by local conditions and neighborhood characteristics, rather than by any single determinant. Direct effects are concentrated in GDP, road density, fiscal expenditure, and altitude, capturing the roles of local economic strength, transport supply, public spending, and topographic constraints. Indirect effects are concentrated in population and fiscal expenditure, showing that accessibility also responds to demographic and fiscal conditions in surrounding counties. Several effects operate in opposing directions. Fiscal expenditure has a negative direct association with local accessibility but a positive indirect association with neighboring accessibility. This contrast is intuitive: counties with high fiscal expenditure relative to GDP are often transfer-dependent and poorly equipped, whereas health services funded in one county may spill over its borders and benefit residents of adjacent counties. Population shows little direct association but a strong negative indirect one, which is consistent with resource allocation based on service-population quotas, where larger population centers absorb a disproportionate share of facilities and investment. In addition, the positive and significant spatial autoregressive coefficient indicates that accessibility moves together across neighboring counties, so improvements in one county are likely to accompany, and be accompanied by, changes in surrounding counties. These combined patterns point to a plausible interpretation: stronger economies and denser road networks support local accessibility; population concentration can drain resources from peripheral counties; and altitude remains a hard constraint that limits both local accessibility and the ability of neighboring regions to compensate. Together these forces help explain the east-high/west-low and core-periphery structure observed in Sichuan. We caution, however, that these are conditional spatial associations rather than causal findings.

It should also be noted that patient flows and actual healthcare-seeking behavior are not directly examined in this study, as no such data are available in our analytical framework. The spatial econometric results therefore provide at best indirect evidence on the underlying mechanisms: the significant indirect effects and spatial lag terms reveal a systematic association between accessibility in one county and the characteristics of its neighbors, a pattern consistent with what would be expected if patients crossed administrative boundaries for care or if resource allocation responded to demand pressures from surrounding areas. Nevertheless, such evidence is insufficient to confirm the mechanisms at work, and future research would benefit from more direct tests using patient-flow data or quasi-experimental designs.

## 4 Discussion

### 4.1 Spatial pattern of accessibility in Sichuan and contributing factors

In summation, hospital accessibility across Sichuan manifests higher values in the east than in the west and in basins than on plateaus, consistent with the healthcare service patterns reported by Zhang Cheng et al. (35). This disparity partly originates from the province’s physiography. Located along the transitional boundary between China’s first and second geomorphological steps, the Hengduan Mountains and the Western Sichuan Plateau form a natural barrier that separates habitable basins from plateau ecological zones. The two regimes differ sharply in population carrying capacity, transportation infrastructure development costs, and resident travel impedance to care; even substantial public health funding cannot easily replicate the service coverage of basin counties in plateau regions. The pattern also reflects China’s hierarchical healthcare resource allocation framework (36), in which resources follow administrative tiers from provincial capitals to prefectural cities and then county seats. Chengdu, as the provincial administrative center, attracts premier medical resources and acts as the province’s healthcare core; on a stronger economic foundation, the Southern Sichuan Economic Zone has developed several intermediate regional medical hubs. In western Sichuan, notably Garze, Aba, and Liangshan, only prefectural capitals possess relatively complete healthcare resources, while remote border townships remain at the bottom of the allocation hierarchy and struggle to serve dispersed populations. Collectively, the spatial configuration of accessibility is consistent with the interplay of environmental constraints, administrative hierarchies, and economic conditions.

### 4.2 Scale, equity, and spatial interpretation

Overall, hospital accessibility in Sichuan varies considerably across space and across administrative units, and the magnitude of this variation grows as the analytical scale becomes finer. Disparities are smallest among prefecture-level cities, larger among counties, and largest among 1 km grid cells, producing a consistent city < county < grid ordering that aligns with the conclusions of Song et al. (37). Part of this widening reflects the mechanics of aggregation. Averaging heterogeneous grid-level values within administrative units smooths internal differences, making larger units appear more equitable than they are. Scale also determines which inequalities remain visible. Equity assessments at the provincial or municipal scale (38, 39) commonly rely on aggregate indicators such as hospital beds or physicians per 1,000 people. These indicators provide useful macro-level information, yet they treat internally heterogeneous areas as uniform and can therefore understate the accessibility gaps that residents of underdeveloped and remote areas actually face. Because they also say little about whether health resources are ultimately used, they can hinder an accurate assessment of health equity in precisely those regions.

These scale effects are not only methodological; they also raise the question of whether spatial findings can be transferred to other settings. Balsa-Barreiro et al. (40) argue that spatial patterns are jointly shaped by scale, context, and heterogeneity, and therefore conclusions drawn at one geographic level or in one context cannot be assumed to hold elsewhere without re-examination. The ordering reported here partially reflects scale effects arising from the aggregation of administrative units, and it may not appear in the same way in other regions. In Sichuan, hospital accessibility is specifically influenced by the geographic barriers of the western plateau, differences in urban development levels, and shortages of primary care services at administrative borders—conditions closely tied to the province’s unique topography, population distribution, administrative boundaries, and health system structure. Consequently, evaluations of health care accessibility should explicitly account for regional heterogeneity and avoid the uncritical extrapolation of area-specific findings, so that conclusions can better reflect actual conditions.

## 5 Conclusion

Drawing on an externally derived GA2SFCA accessibility measure, this study advances a multidimensional assessment of hospital accessibility in Sichuan Province. By using the coefficient of variation and the Palma ratio to quantify equity and employing global and local spatial autocorrelation and an SDM to characterize spatial dependence and associations, the investigation yields the following insights.

First, overall hospital accessibility is moderate and highly uneven rather than uniformly high or low. Spatially, the Panzhihua-Ya’an-Guangyuan corridor separates eastern basins with higher accessibility from western plateaus with lower accessibility. Pronounced disparities persist between the provincial capital and other prefecture-level jurisdictions. Within western Sichuan, sparse demographic distribution and economic underdevelopment constrain the number and capacity of healthcare facilities, while complex topography and deficient transport networks further entrench low accessibility.

Second, the spatial configuration of hospital accessibility corresponds to a combination of environmental constraints, socioeconomic conditions, transport infrastructure, and China’s hierarchical healthcare resource allocation regime.

Third, the equity assessment reveals a scale-dependent gradient: accessibility appears most equitable at the prefecture level, less equitable at the county level, and least equitable at the grid level. Part of this gradient is attributable to spatial aggregation and MAUP, and the indicators describe territorial inequality across spatial units rather than population-experienced inequality. Spatially, hospital accessibility shows robust positive autocorrelation, with hotspots in the Chengdu Plain and the Southern Sichuan Economic Zone and coldspots in the North-Western Sichuan Ecological Demonstration Zone.

Finally, the regression analyses reveal statistically significant spatial associations rather than causal mechanisms. Road density and GDP had positive direct associations with accessibility, whereas fiscal expenditure and altitude had negative direct associations; population size did not show a significant direct association. In the indirect dimension, population was negatively and fiscal expenditure was positively associated with neighboring counties’ accessibility. Total effects were significantly positive for road density and GDP and significantly negative for population and altitude. The positive SDM spatial parameter indicates spatial dependence or feedback among counties. The GM variable measures total fiscal expenditure rather than health-specific expenditure; the corresponding associations should therefore be interpreted cautiously.

## 6 Recommendations

Grounded in the findings above, the recommendations below are intended as policy implications that combine the empirical patterns with the broader literature and regional context. Policy interventions should account for the distinct developmental trajectories and environmental constraints of sub-provincial regions through differentiated resource allocation. In the Western Sichuan Plateau, where altitude is high, population is sparse, and economic resources are fragile, the construction of new large general hospitals should be de-emphasized in favor of mobile medical clinics and telemedicine hubs, while regional healthcare cores are consolidated in prefectural capitals such as Kangding, Markam, and Xichang (41); Second, Chengdu’s advantages should be leveraged through inter-municipal medical consortia and targeted institutional support to peripheral, resource-scarce, ethnic-minority, and poor regions, thereby attenuating the provincial capital’s concentration effect. Third, Panzhihua’s targeted aid to Liangshan Prefecture should continue, while the Southern Sichuan Economic Zone maintains its balanced multi-centric supply advantage. Fourth, planning should rely less on coarse prefecture-level aggregate indicators and more on county-level and sub-county service distributions. Fifth, healthcare facility siting should be planned jointly with public transit and road-network optimization (42, 43), and tertiary hospitals and regional medical centers should be placed at nodes that minimize patient travel impedance. In the alpine and ethnic-minority territories of western Sichuan, rural road upgrading deserves increased investment to counteract terrain-induced accessibility loss. Finally, the provincial fiscal transfer mechanism should be refined by increasing healthcare-specific subsidies to fiscally constrained areas such as the North-Western Sichuan Ecological Demonstration Zone and Liangshan, with safeguards against ecological fund earmarking crowding out health expenditure (44).

## 7 Limitations

Owing to resource constraints, this study used openly available scholarly datasets, so the analysis inherits the limitations of the source data and the analytical framework. First, the source accessibility metric covers the hospital tier only and omits primary care providers such as village clinics and community health centers; this may systematically underestimate accessibility in rural settlements. Second, the source dataset applies an unmodified GA2SFCA model with tier-specific catchments and Gaussian distance decay, without calibrating parameters to heterogeneous healthcare-seeking behavior; travel behavior is therefore simplified. Third, aggregation from 1 km grids to counties and prefectures used unweighted zonal means, so the results are subject to MAUP, and territorial inequality may differ from population-based inequality. Fourth, the use of cross-sectional data limits the strength of causal inference; therefore, the results should be interpreted as plausible analyses rather than definitive causal evidence. Fifth, this study focuses on Sichuan Province, where substantial internal disparities exist in geographical conditions, population distribution, and economic development, which weakens the generalizability of the findings. Future extensions to other regions should fully account for regional heterogeneity. Future research should integrate high-resolution empirical survey data, use population-weighted accessibility and equity measures, distinguish health-specific expenditure from total fiscal expenditure, and test the sensitivity of the spatial results to alternative weights matrices. Framing the Sichuan findings alongside recent work on travel-time accessibility and adaptive spatial planning for healthcare systems further indicates that equity-oriented allocation should be paired with demand-side vulnerability mapping rather than proximity alone.

## Data Availability

The original contributions presented in the study are included in the article/supplementary material, further inquiries can be directed to the corresponding author.

## Footnotes

1
http://www.gscloud.cn/
